# Deep Learning-Based Modeling of Drug–Target Interaction Prediction Incorporating Binding Site Information of Proteins

**DOI:** 10.1007/s12539-023-00557-z

**Published:** 2023-03-26

**Authors:** Sofia D’Souza, K. V. Prema, S. Balaji, Ronak Shah

**Affiliations:** 1 Department of Computer Science and Engineering, Manipal Academy of Higher Education, Manipal, India; 2 Department of Computer Science and Engineering, Manipal Academy of Higher Education, Bengaluru, India; 3 Department of Biotechnology, Manipal Academy of Higher Education, Manipal, India

**Keywords:** Drug–target interaction, Machine learning, Deep learning, Protein–ligand interaction, Sequence alignment

## Abstract

Chemogenomics, also known as proteochemometrics, covers various computational methods for predicting interactions between related drugs and targets on large-scale data. Chemogenomics is used in the early stages of drug discovery to predict the off-target effects of proteins against therapeutic candidates. This study aims to predict unknown ligand–target interactions using one-dimensional SMILES as inputs for ligands and binding site residues for proteins in a computationally efficient manner. We first formulate a Deep learning CNN model using one-dimensional SMILES for drugs and motif-rich binding pocket subsequences of proteins as inputs. We evaluate and compare the proposed deep learning model trained on expert-based features against shallow feature-based machine learning methods. The proposed method achieved better or similar performance on the MSE and AUPR metrics than the shallow methods. Additionally, We show that our deep learning model, DeepPS is computationally more efficient than the deep learning model trained on full-length raw sequences of proteins. We conclude that a beneficial research approach would be to integrate structural information of proteins for modeling drug-target interaction prediction of large datasets for more interpretability, high throughput, and broad applicability.

**Graphical abstract:**

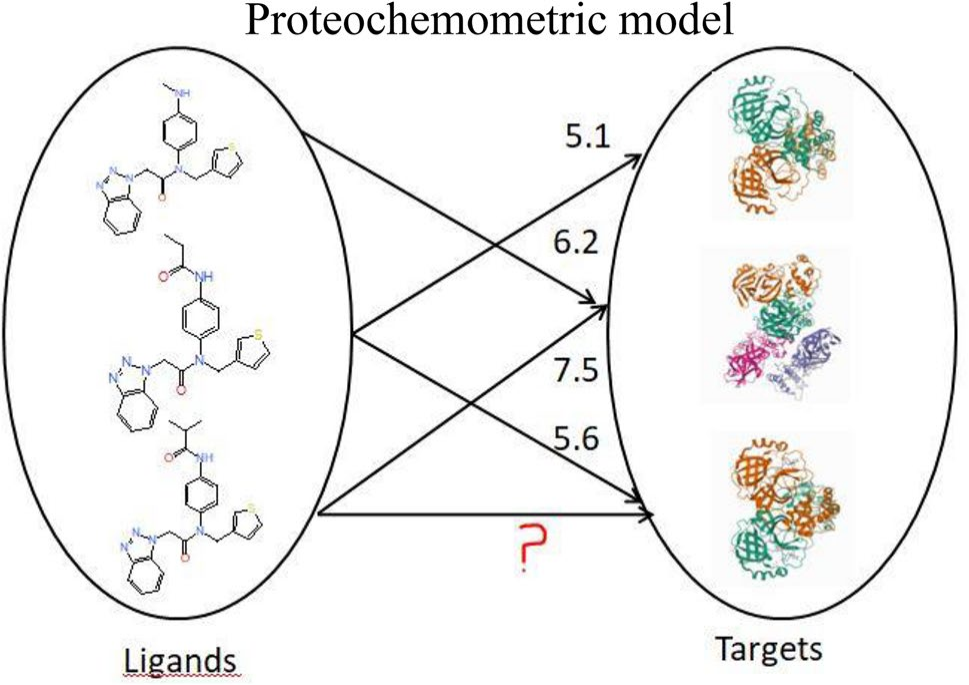

## Introduction

Rapid quantitative prediction of drug-target interaction is essential to drug discovery and development. For decades, the interactions between compounds and proteins were identified by carrying out expensive and time-consuming wet-lab experiments. Drug target interaction prediction is an important task in the drug discovery process, As the chemical space is large, of the order of $10^{60}$ molecules, it is arduous and almost impossible to identify interactions of all the compounds against different targets in the lab. On the other hand, computational screening of drug-target interaction aids in finding a smaller subset of probable candidates which could be taken up for further screening. Computational modeling can help identify side effects of compounds, as a single compound could have effects on multiple targets. Further, the models could be used to identify novel compounds interacting with known targets as well as find an alternate use for existing compounds based on novel interactions. Due to the emergence of high throughput screening, the amount of experimental data available in public databases has significantly increased. The availability of biological data relating to the protein sequence or structure in public databases has also grown tremendously. Chemogenomic models could utilize the available data to predict unknown interactions between proteins and compounds. These models predict the interactions between the ligands and targets by combining information from similar drugs and targets [1]. The models are constructed on the basis of the similarity principle which states that drugs with similar properties interact with similar targets [2].

The Proteochemometric (PCM) or a Chemogenomic model has the advantage of being able to predict interactions between ligands and proteins, even when there is no 3D structure available or when there are a few or no known ligands for the protein [3]. Ligand-based chemogenomic approaches are being pursued in drug discovery as they are computationally less expensive compared to structure-based approaches and can be trained on a large number of available bioactivity data. Consequently, the prediction of interactions greatly enhances the discovery of novel interacting targets and compounds that may find application in drug repurposing efforts [4–7].

Deep learning models such as CNN have shown excellent predictive capability in the field of computer vision. These methods have been used in bioinformatics in genomic studies as well as in models for drug discovery [8, 9]. These models are capable of identifying and learning complex patterns from molecular data [10]. The advantage of a deep learning CNN model is that the raw data can be represented better using non-linear transformations to effectively learn the hidden patterns in the data.

Several authors have studied protein–ligand interaction prediction using machine learning and deep learning techniques. Deep learning models using 3D structures of protein–ligand complexes were developed to predict interactions [11–13]. However, these methods are confined to known protein–ligand complexes. 2D Similarity-based methods using similarities of ligands against similar targets have been employed in predicting interactions. In KronRLS, the authors constructed chemical structure similarity matrices and sequence similarity matrices to represent ligands and proteins. The prediction for each protein–ligand pair is based on the similarity score, which is defined as a Kronecker product of the two matrices [14]. As this method captures only the linear dependencies in the data, a non-linear method, SimBoost, using gradient boosting machine [15] was introduced to predict binding affinity with a prediction interval [16]. In this method, a large number of features were calculated for each protein–ligand pair other than the ligand and the protein features using similarity matrices and constructed features. A deep belief network (DBN) was trained by stacking restricted boltzmann machines (RBM’s) to predict novel DTI’s between approved FDA drugs and targets using Extended-connectivity fingerprints (ECFP) and protein sequence composition (PSC) descriptors [17]. In another study, similar PSC descriptors were used to characterize proteins, and compounds were represented using molecular graph convolution (MGC) to train a scalable neural network model which was compared to the baseline machine learning models, SimBoost and KronRLS [18]. In MDeePred, the proteins were represented using physical, chemical and biological features using CNN to predict compound–protein interactions to achieve significant improvement in prediction performance compared to the baseline methods [19]. A deep LSTM model was used to predict DTIs on four target classes using chemical fingerprints and evolutionary information of proteins [20]. In DeepDTA, the one-dimensional SMILES representation of ligands and raw sequences of proteins were encoded into vector representations using CNN blocks. Further, the combined representations of ligands and proteins were employed to predict interactions. However, the protein sequences were not effectively represented as the model was trained on lengthy sequences [21]. In DeepCDA, the model learned the compound and protein encodings using a combination of CNN and LSTM in the feature encoder layer, which feeds the output to the subsequent layers [22]. The RNN-based encoders, seq2seq were used to encode SMILES of compounds and protein sequences separately in Deepaffinity [23]. The CNN models appended to the RNNs were used to concatenate the outputs of compounds and proteins and fed into more connected layers to predict affinity. The above-discussed machine learning models calculated the similarity matrices of drugs and targets which is computationally expensive. On the other hand, the deep learning models computed a large number of drug and protein descriptors which makes the models less interpretable. The unequal and raw protein sequences were used to model drug-target interaction (DTI) prediction in all the above methods which significantly increased the training time.

The hypothesis in this work is that an interpretable drug–target interaction prediction model could be developed using one-dimensional SMILES as drug descriptors, and protein-binding site subsequences. The prediction could be achieved by incorporating the combined features using a Deep CNN, which has outperformed state-of-the-art machine learning models due to its ability to learn useful patterns from raw data using a hierarchical structure of the deep neural network. The extracted protein subsequences contain useful binding information for representing the contact residues and residues involved in medium-range interactions.

In this paper, we have modeled the compound–protein interaction prediction using a one-dimensional representation of proteins and ligands by training a deep CNN model using the extracted features of proteins as subsequences. The protein subsequences incorporating the binding pocket information of proteins were used as input instead of the raw sequences. The method uses the one-dimensional features of drugs and proteins and does not require the 3D structures as inputs to the model. It is possible to develop predictive models by using the amino acid residues of the binding site where structural information of proteins is available [24]. As many structures of proteins are available, the structural information of the binding domain of proteins was utilized to obtain motif-rich binding site residues lining the binding pocket. If the protein 3D structure is unavailable, ligand-binding sites could be predicted using different sequence-based tools. Unlike the above-discussed models, our models are trained on shorter protein sequences using a hybrid approach by incorporating structural information of protein binding sites.

The main contributions of the paper are as follows:-

- Proposing a better representation of proteins by considering residues of the binding pocket.
- Improving the training time of the prediction model due to shorter protein sequences.
- Compared our proposed model with the state-of-the-art deep learning model using training epochs as an additional metric.

## Materials and Methods

### Dataset

**Fig. 1:**
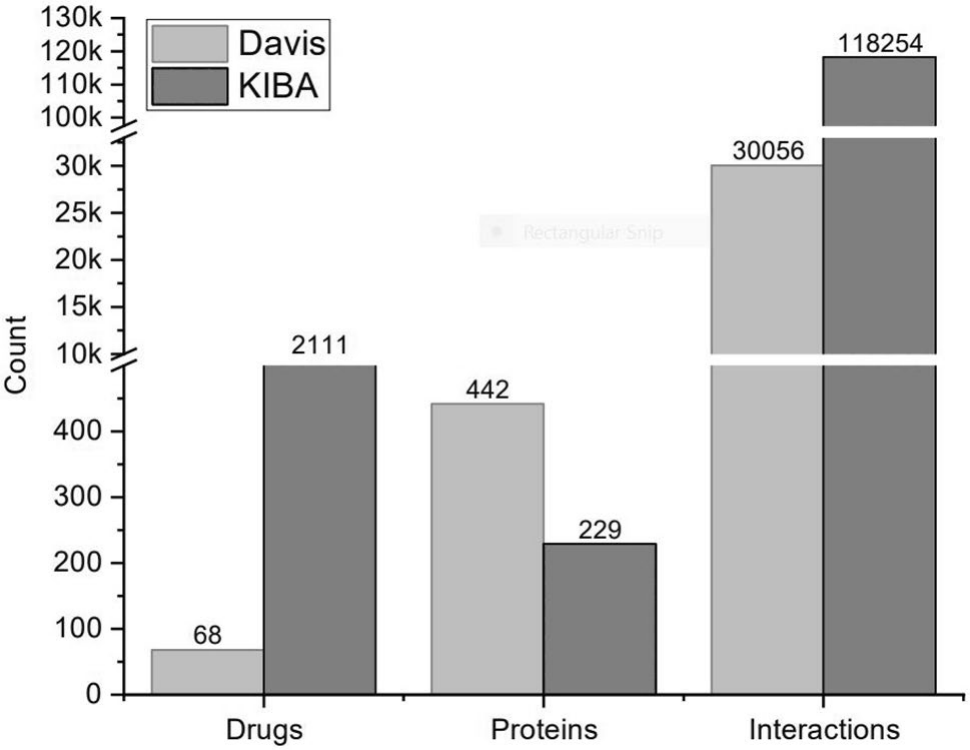
Composition of DAVIS and KIBA Datasets

The composition of ligands, proteins, and interactions of the benchmark datasets, Davis and KIBA, is shown in Fig. 1. The bioactivity values of Davis and KIBA datasets were converted to $Pk_d$ and $PIC_{50}$, as described in the previous literature (Table 1). For a fair comparison with the earlier methods, we divided the datasets into six equal parts. One part was taken as an independent test set. The remaining five parts were used for tuning the hyper-parameters through five-fold cross-validation.

**Table 1:** Dataset statistics

	Davis	KIBA
Datapoints	30,056	1,18,254
Sequences	442	2111
Ligands	68	229
Bioactivity	$Pk_d$	$PIC_{50}$

### Representation of Drugs

**Fig. 2:**
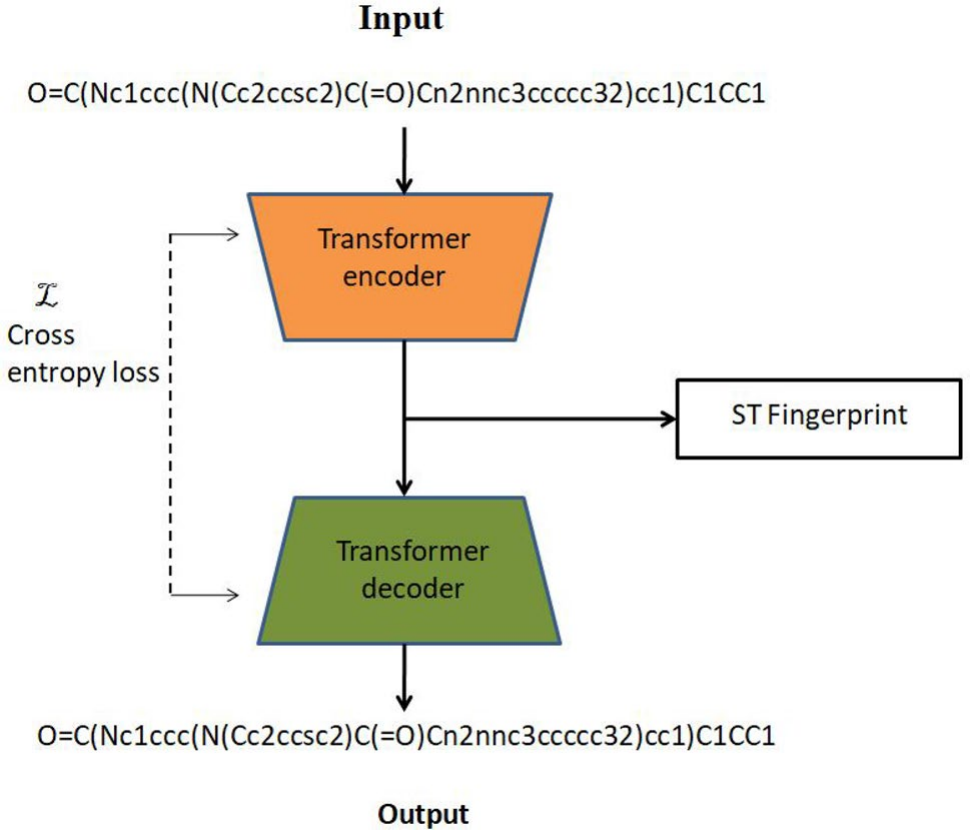
Transformer encoder–decoder network using SMILES. The generated ST Fingerprint was used as input to the molecule encoder of the DeepPS (FP) model

The molecules, represented as a one-dimensional SMILES notation [25] were encoded using CNN. The integer encodings were used to represent characters of SMILES comprising 64 labels. The integer encoded SMILES strings were given as input to the molecule encoder in the DeepPS model. However, in the DeepPS (FP) model, the one-dimensional SMILES strings of the molecules were used to generate fingerprints using the SMILES transformer, as shown in Fig. 2. The SMILES transformer comprised the encoder–decoder network with four transformer blocks each. Each block has four-head attentions with 256 embedding dimensions, and two linear layers [26]. The pre-trained SMILES transformer [27], trained on unlabelled SMILES, was used to generate ST Fingerprints. The symbol-level representations from each of the four transformer blocks were pooled together to obtain ST fingerprints. The fingerprints generated were 1024 bits for each molecule. The ST Fingerprints were used as input to the molecule encoder in the DeepPS (FP) model.

### Feature Selection of Proteins

The Davis [28], and KIBA [29] datasets of kinases consists of the bio-activity data of typical and atypical kinases. A structure-based approach can understand the similarity and dissimilarity of both types of kinases’ conserved regions. The conservation and variation of residues of the ATP binding pocket and the region in the vicinity of this pocket were studied using active-conformation structures [30]. The structure-based binding site alignment of conserved regions of highly similar kinases reveals the presence of common structural elements such as secondary structures and functional motifs such as “DFG” and “HRD” [31]. Most of the conserved regions are aligned. The unaligned blocks contain specific insertions of varying lengths in between in some kinases. Besides, some kinases have shifted secondary structures. Various types of inhibitors bind to the proteins at different binding sites. In typical kinases, the binding site consists of secondary structural elements and functional motifs present in the protein kinase domain. The key regions which are associated with the binding of inhibitors are the HRD motif, DFG motif, G-rich loop, alphaC-helix, catalytic and activation loops [32–34] To identify the binding domain, the protein sequences of the kinases in the datasets were extracted from Uniprot [35].

**Fig. 3:**
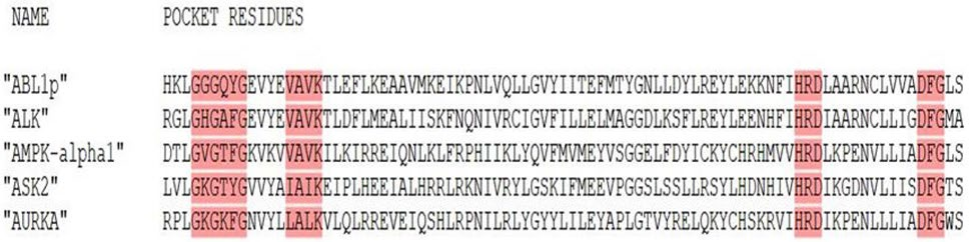
Examples of the binding pocket residues with highlighted motifs selected as protein features

The binding sites of protein kinases contain specific motifs which are rich in information attributing to kinase specificity. Identifying the conserved regions that contribute to the specificity of kinases and representing them to be amenable for modeling can provide better predictive capability and interpretation. The varying amino acids in the conserved regions contribute to the specificity as the binding region is highly conserved in kinases. The binding site residues obtained from the catalytic cleft of kinases enable the comparison of the interaction pattern of kinase inhibitors. The binding pocket residues of all protein kinases present in the datasets were extracted using the binding site positions from the sequences after performing sequence alignment of the structural elements implicated in the binding process [36]. All the protein subsequences of the binding pocket comprised the G-rich loop, alphaC-helix, catalytic loop, and motifs such as VAIK/VAVK motif, HRD motif, and the DFG motif and seem to be aligned to the respective motif positions, except for some atypical kinases which had missing or differing secondary structure elements. The fixed length of 85 binding pocket residues was obtained for each of the proteins (Fig. 3). The protein subsequences containing the binding pocket residues were used as input to the protein encoder in the DeepPS and DeepPS (FP) models.

### Proposed Chemogenomic Model

**Fig. 4:**
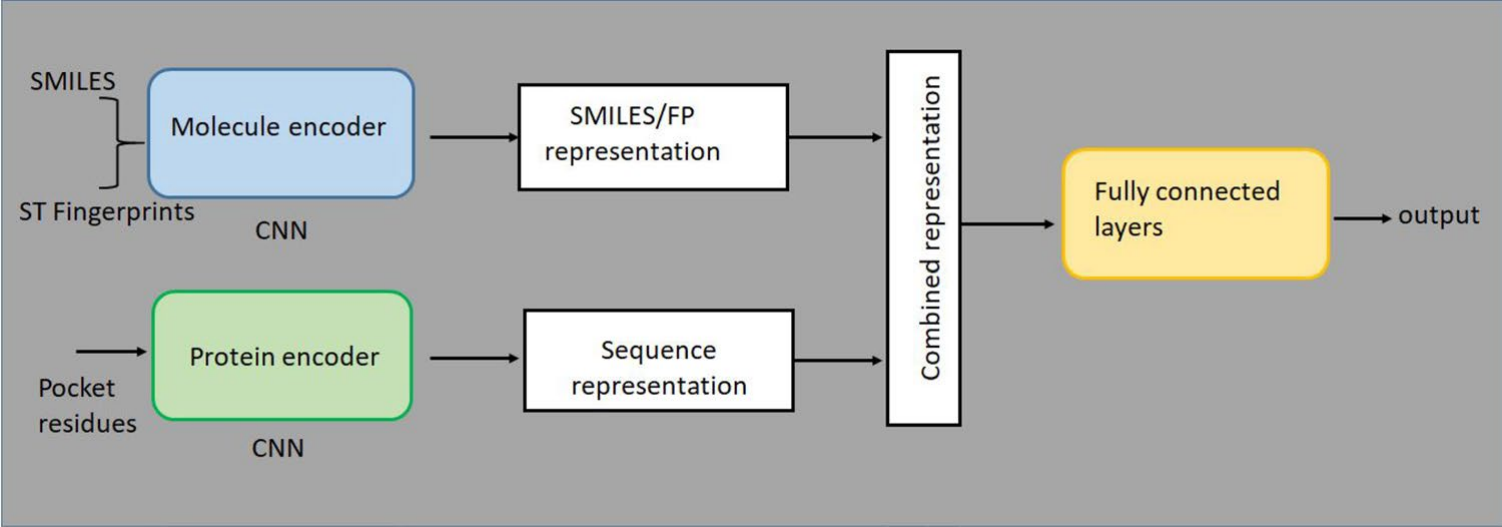
CNN-based Chemogenomic model with SMILES and binding pocket residues as inputs for drugs and proteins (DeepPS). The inputs for drugs in DeepPS (FP) are ST Fingerprints and binding pocket residues for proteins )

The proposed CNN-based chemogenomic models with deep learning contain four main building blocks. The first block is the molecule encoder that encodes the SMILES strings of ligands in the DeepPS model and ST Fingerprints in the DeepPS (FP) model. The SMILES strings were represented using integer encodings to represent unique letters. The Transformer–encoder–decoder network was utilized to generate SMILES transformer fingerprints (ST fingerprints)in DeepPS (FP) model. Second, the protein encoder embeds the features from the pocket residues given as input using label encodings to represent 26 categories. The pocket residues are used as inputs for proteins in both DeepPS and DeepPS (FP) models. The SMILES and proteins were of different lengths for both datasets. The fixed length of 85 and 100 were chosen for SMILES of Davis and KIBA datasets, respectively, while the length of the protein subsequences was fixed at 85 for both datasets for effective representation. The outputs from the molecule encoder and the protein encoders were concatenated and given as input to three fully connected layers of the CNN with dropout layers in between them. The dropout layers are used to reduce the overfitting of the data. The final CNN output layer predicts the outputs. The architecture, along with the building blocks, is shown in Fig. 4.

## Results

The baselines evaluated in our experiments are the KronRLS, SimBoost, and DeepDTA. The DeepPS model was trained on the SMILES and binding pocket residues. The second model, DeepPS (FP) was trained on Smiles transformer fingerprints and binding pocket residues.

**Table 2:** Parameter settings for CNN

Parameters	Range
No. of filters	32*1; 32*2; 32*3
Length of filter (compounds)	[4, 6, 8]
Length of filter (proteins)	[4, 8, 12]
Hidden neurons	1024; 1024; 512
Batch size	256
Dropout	0.1
Optimizer	Adam
Learning rate	0.001

We evaluated the performance of our method on benchmark datasets Davis and KIBA. The same settings for the train and test folds were used as given in the literature [21] for a fair comparison. The entire dataset was divided into six folds, out of which one fold was used as an independent test set. The remaining folds were used for training using nested cross-validation to obtain tuned hyper-parameters. The parameter settings used for the CNN model are as given in Table 2. The maximum sequence length of the proteins for the models was set to 85 for both datasets as only 85 residues are involved in binding. An early stopping strategy using validation mean squared error (MSE) as a performance measure was adopted to avoid overfitting of the model during training.

### Evaluation Metrics

In this study, we used four evaluation metrics, MSE, CI, $r_m^2$, and Area under precision-recall (AUPR). The evaluation metrics other than AUPR were used to evaluate continuous regression outputs. AUPR was obtained by binarising the regression outputs based on the threshold value. A threshold value of 7 was chosen for the Davis dataset and 12.1 for the KIBA dataset according to the previous work [37].

Concordance index (CI) was utilized to measure the effectiveness of the model with continuous outputs [38]. It measures the probability of the similarity between the actual values and the predicted values of two random protein–ligand pairs.

1 $$\begin{aligned} CI= \frac{1}{Z} \sum _{\delta _i > \delta _j} h(m_i - m_j) \end{aligned}$$

where $m_i$ is the prediction value for the greater affinity $\delta _i$, $m_j$ is the predicted value for the smaller affinity $\delta _j$, *Z* is the normalization constant that equals the number of data pairs with different label values and *h*(*x*) is the step function defined as

$$\begin{aligned} \text{ h }(x)=\left\{ \begin{array}{rl} 1 &{} \text{ if } x>0 \\ 0.5 &{} \text{ if } x=0 \\ 0 &{} \text{ if } x<0 \end{array} \right. \end{aligned}$$

Mean squared error is defined as

2 $$\begin{aligned} MSE = \sum ^{\infty }_{i=1}h(y_i - y_j)^2 \end{aligned}$$

The external predictive power of the model is given by $r_m^2$ metric, which is defined as follows.

3 $$\begin{aligned} {r_m}^2 = r^2 \times (1-\sqrt{r^2-r_o^2}) \end{aligned}$$

where $r_o^2$ is the squared correlation coefficient without intercept, $r^2$ is the squared correlation coefficient with intercept.

The area under the precision-recall (AUPR) curve assesses a binary model by taking the average of the precision values across all recall values. The AUPR method is suitable for estimating the accuracy of datasets having imbalanced classes with skewed distribution [39]. The thresholds for binarising the outputs were chosen as proposed by He et al. [16].

In addition to these metrics, our models were evaluated on training time as an additional metric to gain insights on the model training in order to avoid overfitting of the model.

### Comparison of Shallow and Deep Learning Models

The results of our chemogenomic models were compared with the baseline machine learning shallow methods, KronRLS and SimBoost, and the deep learning method, DeepDTA. The models were compared against shallow methods as these methods were trained on computed features. The results obtained by applying our method on Davis and KIBA datasets were evaluated on average mean squared error (MSE) and average Concordance index (CI) over the independent test set. The results on Davis and KIBA datasets are presented in Tables 3 and 4. The results obtained by the shallow methods have been taken from the literature. The code for the deep learning method DeepDTA was downloaded and run in our setting for comparison.

**Table 3:** The average Concordance Index and Mean squared error scores of the test set of Davis dataset for the compared methods

	Proteins	Compounds	CI	MSE
KronRLS	S-W	Pubchem Sim	0.871 (0.0008)	0.379
SimBoost	S-W	Pubchem Sim	**0.872 (0.002)**	**0**.**282**
DeepDTA	CNN	CNN	0.851 (0.004)	0.379
DeepPS (FP)	CNN	Tran-CNN	0.861(0.007)	0.375
DeepPS	CNN	CNN	0.854(0.007)	0.353

For every metric, the value for the best performing method has been highlighted in bold font

In the Davis dataset, our models have achieved comparable performances on the MSE and CI values against the other methods. Even though SimBoost has slightly better performance than DeepPS, our model is scalable and performs better than SimBoost on time and space complexity metrics as SimBoost requires computationally expensive matrix factorization as it relies on similarity matrices. The DeepPS model has achieved better performance than DeepPS (FP) as some information could have been lost in the generation of fingerprints.

**Table 4:** The average Concordance index and Mean squared error scores of the test set of KIBA dataset for the compared methods

	Proteins	Compounds	CI	MSE
KronRLS	S-W	Pubchem Sim	0.782 (0.0009)	0.411
SimBoost	S-W	Pubchem Sim	0.836 (0.001)	0.222
DeepDTA	CNN	CNN	0.765(0.002)	0.375
DeepPS (FP)	CNN	Tran-CNN	0.782(0.003)	0.310
DeepPS	CNN	CNN	**0.844(0.003)**	**0**.**218**

For every metric, the value for the best performing method has been highlighted in bold font

On the KIBA dataset, the performance of the DeepPS model is better than all the models on the CI and MSE metrics. The KIBA dataset consists of more proteins and interaction data as compared to the Davis dataset resulting in better generalization.

The external predictivity of the model on an independent test set was analyzed using the $r_m^2$ metric [40]. The values obtained for our models were greater than 0.5 for both Davis and KIBA datasets indicating that the models were acceptable. The standard deviations are given in parenthesis. The AUPR values were calculated by binarising the outputs. A threshold ($Pk_d \ge 7$) was set for the Davis dataset to classify as binding. For the KIBA dataset, the value was set to 12.1. The results of the $r_m^2$ and AUPR metrics for both datasets are summarized in Tables 5 and 6.

**Table 5:** The average $r_m^2$ and AUPR scores of the test set for the Davis dataset

	Proteins	Compounds	$r_m^2$(std)	AUPR(std)
KronRLS	S-W	Pubchem Sim	0.407 (0.005)	0.661 (0.010)
SimBoost	S-W	Pubchem Sim	**0.644 (0.006)**	0.709 (0.008)
DeepDTA	CNN	CNN	0.526 (0.017)	0.567 (0.010)
DeepPS (FP)	CNN	Tran-CNN	0.573(0.005)	0.681(0.005)
DeepPS	CNN	CNN	0.546(0.003)	**0.710(0.003)**

For every metric, the value for the best performing method has been highlighted in bold font

**Table 6:** The average $r_m^2$ and AUPR scores of the test set for the KIBA dataset

	Proteins	Compounds	$r_m^2$ (std)	AUPR (std)
KronRLS	S-W	Pubchem Sim	0.342 (0.001)	0.635 (0.004)
SimBoost	S-W	Pubchem Sim	**0.629 (0.007)**	0.760 (0.003)
DeepDTA	CNN	CNN	0.458 (0.009)	0.582 (0.004)
DeepPS (FP)	CNN	Tran-CNN	0.517(0.005)	0.691(0.002)
DeepPS	CNN	CNN	0.604(0.003)	**0.762(0.004)**

For every metric, the value for the best performing method has been highlighted in bold font

**Fig. 5:**
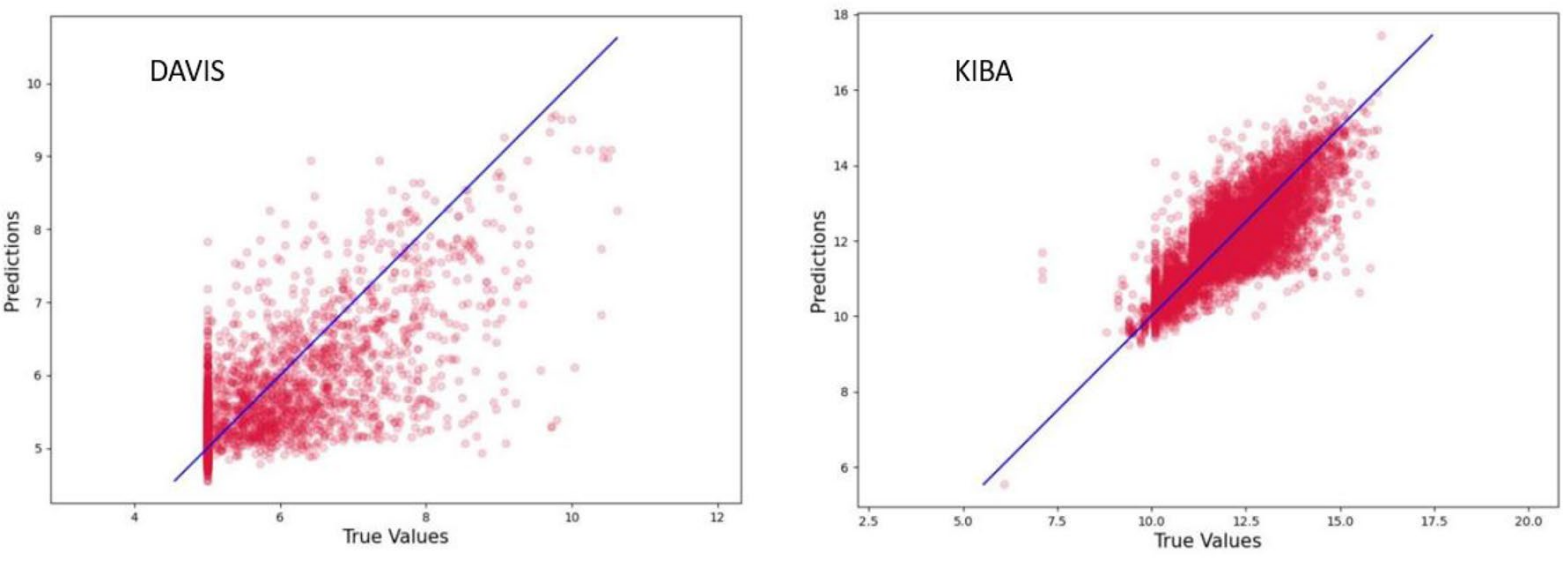
Binding affinity prediction of DeepPS model.** a** experimental values versus predicted values for Davis dataset.** b** experimental values versus predicted values for KIBA dataset

The excellent correlation of the predictions obtained by different input representations and methods employed removes the chance correlation and emphasizes the predictive power of the models developed. The predicted versus actual plots obtained from the DeepPS model on Davis and KIBA datasets are shown in Fig. 5. The Davis dataset has less diverse ligands compared to the KIBA dataset. The data points are aggregated around the regression line in KIBA dataset compared to the Davis dataset.

Based on the obtained results, it could be inferred that the binding between proteins and ligands depends not only on the binding pocket residues but also on residues outside the binding pocket that can have long-range effects. The flexibility of the protein and conformational adjustment during the binding process also contributes to the binding as adjacent pockets may also be involved in binding [41]. Our model based on the binding pocket residues achieved better or comparable results than shallow methods on MSE and CI metrics on both datasets suggesting that the motif-rich features representing the binding pocket were able to capture the physicochemical properties of the pocket. The motif-rich subsequences are part of the secondary structural elements of the proteins interacting with the ligands that contain the necessary binding features. Predicting novel interactions between ligands and proteins in drug discovery is more important than missing them out. In other words, false negatives should be minimized as false positives do get checked during wet-lab experiments. To achieve this, our model is computationally efficient but slightly less accurate for large-scale binding affinity prediction compared to other deep models trained only on raw sequences and SMILES strings.

### Performance Evaluation of the Deep Learning Models on Davis and KIBA Datasets

**Table 7:** Comparison of metrics with baseline Deep learning model

Dataset	Method	Specificity	Sensitivity	AUROC
Davis	DeepDTA	0.98	0.36	0.67
	DeepPS	0.99	0.24	0.62
KIBA	DeepDTA	0.93	0.64	0.78
	DeepPS	0.93	0.65	0.79

For evaluating our model’s performance, various metrics such as specificity, sensitivity, and accuracy were also computed by taking the thresholds from the generated regression outputs. The DeepPS model achieved slightly better performance compared to DeepDTA on the KIBA dataset and slightly lower values on the Davis dataset (Table 7). The low values could be attributed to the protein features included in the model. As the Davis dataset consists of a lesser number of proteins and interactions compared to the KIBA dataset, the model may not have been able to completely capture the patterns in the data. Also, as the binding affinity between drugs and targets depends on the local and non-local interactions, including distant amino acid residues contributing to the non-local interactions may improve model performance [42].

**Fig. 6:**
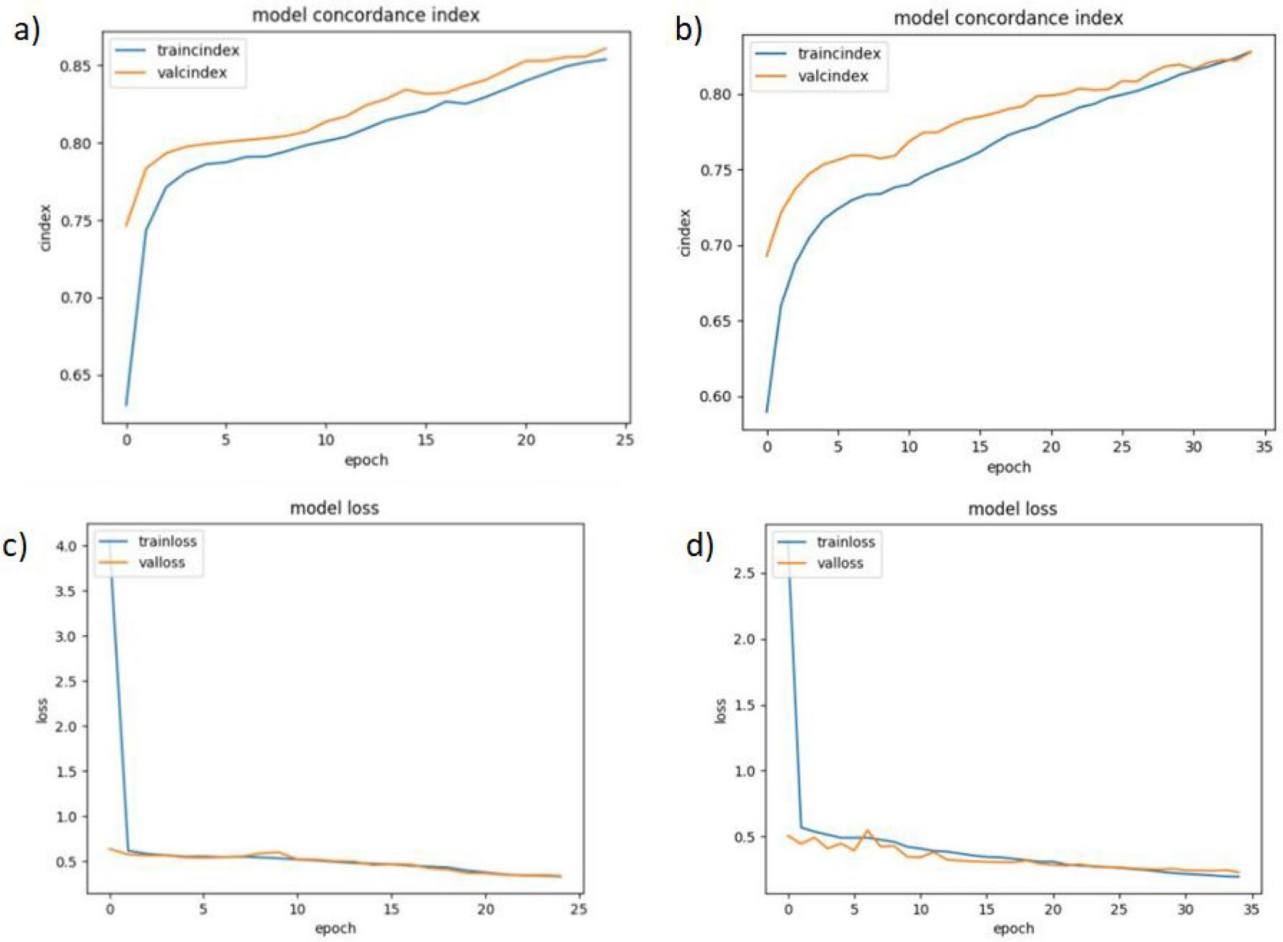
Training results of DeepPS model. **a** CI plot for validation set and training sets of Davis dataset. **b** CI plot for validation set and training sets of KIBA dataset. **c** Plot of MSE for validation set and training sets of Davis dataset. **d** Plot of MSE for validation set and training sets of KIBA dataset

The training time of a model is proportional to the size of the inputs. Our best performing model, DeepPS, was evaluated on training time with the DeepDTA method taken as a baseline for comparison. The DeepDTA method was chosen as a baseline deep learning model as our models employed CNN blocks for encoding drug and protein features similar to DeepDTA. The plots of average CI and MSE metrics on the training sets for the Davis and KIBA datasets of the DeepPS are displayed in Fig. 6. The DeepPS model shows considerable improvement in training time, as seen by the fewer epochs. The learning of DeepPS was completed in 25 epochs and 35 epochs for Davis and KIBA datasets, respectively. The concordance index curves and the loss curves for the training and validation set indicate that the model is not overfitting. The curves show a good fit as the validation gap is reduced to the point of stability (epochs) and has a small gap with the training curves. The training of the DeepDTA model was completed in 100 epochs for both datasets, which is longer. The improvement in training time of DeepPS could be attributed to the shorter sequence length of the proteins.

Further, we compared the execution time taken by our method against DeepDTA on the Davis and KIBA datasets. The results are summarized in Table 8.

**Table 8:** The approximate training time (in hours) of Davis and KIBA datasets

Method	Dataset	Pre-processing	Training time
DeepPS	Davis	2	4
	KIBA	3	7
DeepDTA	Davis	NIL	10
	KIBA	NIL	15

The DeepPS and DeepDTA were executed on Google Colab cloud platform on a GPU machine. The starting and completion times were recorded. On comparing the training times, we can see that DeepPS is able to complete the job faster compared to DeepDTA mainly because of the pre-processing step. Further optimization could be achieved if the pre-processing step is incorporated in the DeepPS algorithm instead of a executing it as a separate script. The pre-processing involved aligning the protein structures to obtain the binding amino acids. The pre-processing step was carried out on a standalone machine.

## Conclusion

An understanding of the important features contributing to the predictive performance of the model is important for model optimization. However, as deep learning models are considered black boxes as it is not easy to understand the contributing features, we tried to optimize the neural network model by extracting the relevant protein features and combining them with the drug features. The proposed deep learning-based method predicts drug-target interactions using only one-dimensional SMILES strings of drugs and protein subsequences obtained from binding pocket information thereby proving our hypothesis. The CNN blocks were used for encoding one-dimensional descriptors of drugs and proteins. Further, our model trained on the binding site residues achieved comparable performance to the baseline shallow methods and is computationally efficient than the baseline machine learning models as it does not require the construction of similarity matrices. This study also offers additional confidence to the previous works on these datasets to generalize using a hybrid chemogenomic approach for computationally efficient drug-target interaction prediction compared to other approaches while offering comparable performance values. Our findings could be used to model drug-target interactions to find side effects that could be used in drug repurposing efforts. Finally, this work provides a faster, rational, and straightforward predictive model that may be employed to guide future experiments in drug discovery.

## Data Availability

The data used to support the findings of this study are available from the corresponding author upon request.
